# Underwater endoscopic submucosal dissection with gel immersion and red dichromatic imaging for an anastomotic lesion following colon surgery

**DOI:** 10.1055/a-2612-3150

**Published:** 2025-06-18

**Authors:** Takahiro Muramatsu, Masakatsu Fukuzawa, Midori Mizumachi, Satoshi Shimai, Yuki Yamamoto, Reisuke H. Takahashi, Takao Itoi

**Affiliations:** 1 38548 Department of Gastroenterology and Hepatology, Tokyo Medical University Hospital, Tokyo, Japan; 2 38548 Department of Diagnostic Pathology, Tokyo Medical University Hospital, Tokyo, Japan

Endoscopic submucosal dissection (ESD) of lesions involving colorectal surgery anastomosis can be challenging because of severe fibrosis as well as the presence of staples and suture lines 1 . Traction or underwater ESD (U-ESD), may help to address these difficulties 2 3 , but this strategy is still emerging. Recently, it has been reported that gel immersion during ESD improves the visual field 4 , and red dichromatic imaging (RDI) increases the visibility of the submucosal layer 5 . Herein, we report a case of U-ESD involving a combination of gel immersion and RDI for an anastomotic lesion following colon surgery ( Video 1 ).

Underwater endoscopic submucosal dissection with gel immersion and red dichromatic imaging to treat an anastomotic lesion following colorectal surgery.Video 1

The patient was an 83-year-old man who had undergone right hemicolectomy to treat cancer of the ascending colon four years prior. Surveillance colonoscopy revealed a flat, elevated lesion (20 mm, type 0–IIa) above the anastomosis that was considered to be a high-grade adenoma or intramucosal carcinoma ( Fig. 1 ). ESD was therefore planned. Because of poor scope maneuverability, U-ESD was initially attempted. However, the field of view was poor because of intestinal residuals, so gel was used to obtain a clear view. After forming a mucosal flap, a whole circumferential incision was made ( Fig. 2 **a–h** ). Once we switched from white-light imaging to RDI, the submucosal layer became transparent, the staples were visible, and the appropriate incision line could be identified ( Fig. 3 ). Submucosal dissection was continued and the lesion was mostly resected; however, minor portions remained, so additional resection was performed via underwater endoscopic mucosal resection. The resected surface was closed using clips ( Fig. 2 **i–o** ). The pathological diagnosis was high-grade tubulovillous adenoma ( Fig. 4 ).

**Fig. 1:**
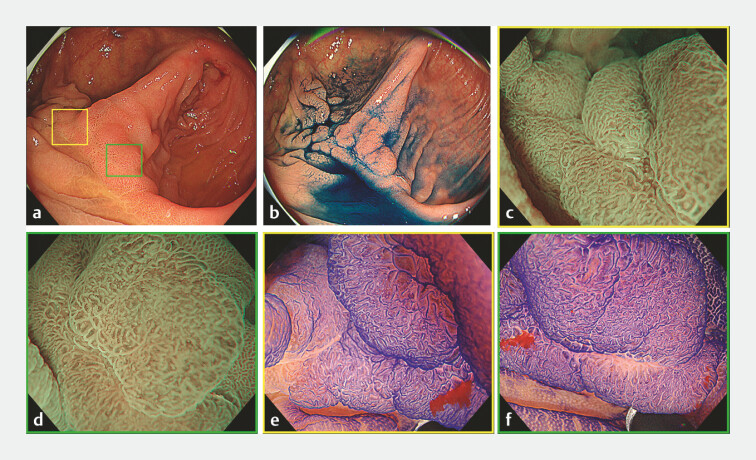
Endoscopic images. **a** White light image. Lower gastrointestinal endoscopy revealed a flat, elevated lesion (0–IIa) located on the anastomosis after right hemicolectomy (lesion diameter: 20 mm). **b** Image with indigo carmine. The lesion straddled the ileal and colonic sides. **c** Magnified narrowband imaging (NBI) view of the yellow square in **a** . Irregular microvessels and surface patterns were observed, corresponding to Japan NBI Expert Classification (JNET) type 2B. **d** Magnified NBI view of the green square in **a** . This area also showed JNET type 2B. **e** Magnified endoscopy with crystal violet staining of the yellow square in a revealed a VI pit pattern. **f** Magnified endoscopy with crystal violet staining of the green square in a also showed a VI pit pattern.

**Fig. 2:**
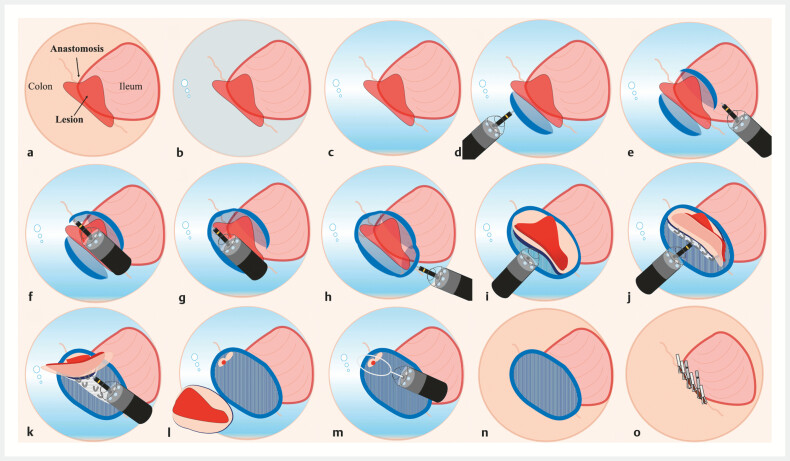
Schematic diagram of underwater endoscopic submucosal dissection with gel immersion to treat an anastomotic lesion. **a** Under gas view. **b** Underwater view. **c** Immersed views in water and gel. A clearer view was obtained following gel injection. **d** The initial mucosal incision was performed from the colonic side to make a mucosal flap. **e** A mucosal incision was also made on the ileal side of the lesion. **f** A mucosal incision was made on the distal suture line. **g** The mucosal incision was widened. **h** A mucosal incision was made on the proximal suture line. **i** Slipping into the submucosal layer. **j** The submucosal layer with fibrosis above the staples was dissected. **k** The remaining submucosal layer was dissected. **l** A small amount of lesion and normal mucosa remained. **m** Underwater endoscopic mucosal resection was used to remove the few residual lesions. **n** The resected surface. **o** The mucosal defect was closed using conventional and mantis-like clips.

**Fig. 3:**
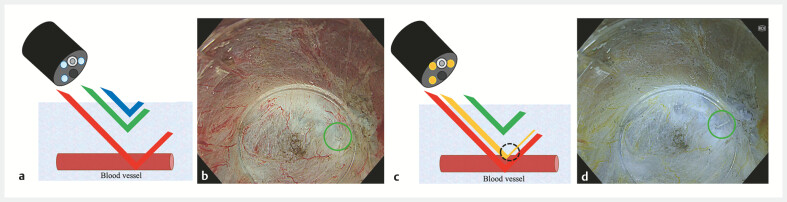
Comparison of white-light imaging (WLI) and red dichromatic imaging (RDI). **a** Schematic diagram of WLI, which uses blue, green, and red light. **b** Endoscopic image of WLI. The staple was difficult to identify (green circles). **c** Schematic diagram of RDI, which uses green, amber, and red lights. If blood vessels are present in the deeper layers, amber is strongly absorbed and less light returns to the surface layer, forming a contrast (black dotted circle). **d** Endoscopic image of RDI. Comparing WLI and RDI, the submucosal layer appeared to be more transparent under RDI and the staple was more visible (green circles).

**Fig. 4:**
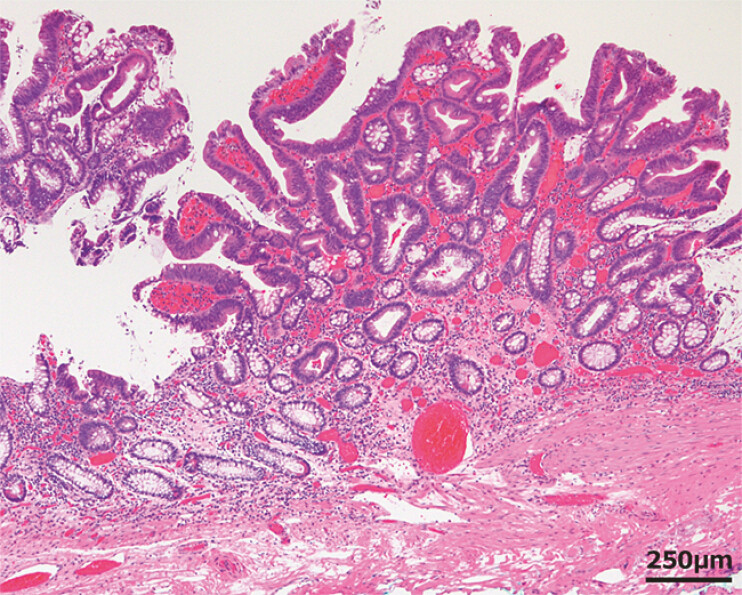
Histopathological images of the resected specimens. The pathological diagnosis was high-grade tubulovillous adenoma. The horizontal margin was considered inconclusive, and the vertical margin was considered negative.

This approach facilitates safe ESD for colorectal anastomotic lesions by improving visibility and maneuverability using water and gel immersion, allowing the most appropriate incision line in the fibrotic area to be identified via RDI.

Endoscopy_UCTN_Code_TTT_1AQ_2AI

## Notes

Endoscopy E-Videos https://eref.thieme.de/e-videos
*E-Videos* is an open access online section of the journal *Endoscopy* , reporting on interesting cases and new techniques in gastroenterological endoscopy. All papers include a high-quality video and are published with a Creative Commons CC-BY license. Endoscopy E-Videos qualify for HINARI discounts and waivers and eligibility is automatically checked during the submission process. We grant 100% waivers to articles whose corresponding authors are based in Group A countries and 50% waivers to those who are based in Group B countries as classified by Research4Life (see: https://www.research4life.org/access/eligibility/ ). This section has its own submission website at https://mc.manuscriptcentral.com/e-videos .
